# How much do combined affective and cognitive impairments worsen rehabilitation outcomes after hip fracture ?

**DOI:** 10.1186/s12877-018-0763-x

**Published:** 2018-03-12

**Authors:** Laurence Seematter-Bagnoud, Sylvain Frascarolo, Christophe J. Büla

**Affiliations:** 1 Service of Geriatric Medicine and Geriatric Rehabilitation, Department of Medicine, Lausanne University Hospital, Mont Paisible 16, 1011 Lausanne, Switzerland; 2 Institute of Social and Preventive Medicine, Lausanne University Hospital, Lausanne, Switzerland

**Keywords:** Hip fracture, Rehabilitation, Functional status, Elderly persons, Mental status

## Abstract

**Background:**

To investigate the association between isolated and combined affective and cognitive impairments with functional outcomes and discharge destination in older patients admitted to rehabilitation after a hip fracture.

**Methods:**

Prospective study in 612 community-dwelling patients aged 65 years and over, admitted to rehabilitation after surgery for hip fracture. Information on socio-demographics, medical, functional, affective, and cognitive status was systematically collected at admission. Functional status, length of stay and destination were assessed at discharge. Functional improvement was defined as any gain on the Barthel Index score between admission and discharge from rehabilitation.

**Results:**

At admission, 8.2% of the patients had isolated affective impairment, 27.5% had cognitive impairment only, and 7.5% had combined impairments. Rate of functional improvement steadily decreased from 91.2% in patients with no cognitive nor affective impairment to 73.8% in those with combined impairments. Compared to patients without any impairment, those with combined impairments had lower odds of functional improvement, even after adjustment for age, gender, health and functional status at admission (adjOR: 0.40; 95%CI: 0.16–1.0; *p* = .049). The proportion of patients discharged back home gradually decreased from 82.8% among patients without any impairment to only 45.6% in patients with combined impairments. In multivariate analysis, the odds of returning home remained significantly reduced in these latter patients (adjOR: 0.31; 95%CI:0.15–0.66; *p* = .002).

**Conclusions:**

Affective and cognitive impairments had both independent, and synergistic negative association with functional outcome and discharge destination in patients admitted to rehabilitation after a hip fracture. Nevertheless, patients with combined affective and cognitive impairments still achieved significant functional improvement, even though its magnitude was reduced. Further studies should investigate whether these patients would benefit from better targeted, longer, or more intensive rehabilitation interventions to optimize their functional recovery.

## Background

Hip fracture is a major threat to an older person’s independence. Overall, one in four hip fracture patients will have permanent lower-body disability as a result of the fracture [1]. About 40 to 70% of hip fracture patients do not regain their pre-fracture functional status [2–4]. As a result, most will subsequently require a higher level of formal home care and about 15 to 30% will not be able to return to their own home, but to a nursing home [5–7]. Prolonged stays in acute and post-acute care hospitals, as well as additional cost linked to increased need for formal care make hip fracture the most costly osteoporotic fracture and a major burden for the health care system [8–10].

Several previous studies investigated the association between patients’ characteristics and functional outcome after rehabilitation following hip fracture surgery. Besides older age, comorbidity, poor nutritional status, and low pre-fracture functional status, cognitive as well as affective impairments have also been associated with poorer functional recovery and increased risk of long term care admission [7, 11–17]. However, some uncertainty remains about the effect of cognitive and affective status. Altogether, studies tend to suggest a negative impact of depressive symptoms and cognitive impairment on rehabilitation outcomes. Results are however heterogeneous, likely because of different study designs and adjustment variables [17–22]. Very few studies tried to disentangle the independent as well as combined impact of cognitive and affective impairments: results suggest that isolated cognitive or affective impairment reduces the likelihood of functional improvement, or its magnitude, with an amplified impact when combined. However, previous studies relied on small sample sizes and used different functional outcomes, such as mobility, activities of daily living, or Barthel Index score, along with different time frames to measure improvement [15, 16].

Therefore, the aim of this study was to examine the isolated and combined effect of cognitive and affective impairments on in-hospital functional improvement, cost of rehabilitation, and discharge destination in patients admitted to rehabilitation after surgery for a hip fracture.

## Methods

### Study population

Study subjects were community-dwelling patients (*N* = 5373) aged 65 years and over who were transferred from an acute ward (mean length of acute care stay: 13 days [23]) to post-acute rehabilitation in an academic medical center in Switzerland between 2002 and 2010. Among those, a total of 665 patients were admitted to rehabilitation after surgery for hip fracture (inter, sub-trochanteric, and femoral neck fractures) and were selected for further specific analyses.

The study was approved by the university review board.

Besides medical and nursing care, patients admitted to this unit usually receive daily physical therapy sessions, as well as occupational therapy twice a week on average.

### Data collection

Within 48 h of admission to rehabilitation, patients were systematically assessed by a nurse and a physician to collect information on socio-demographics, medical, functional, affective, and cognitive status. Pre-fracture performance in Katz’ Basic Activities of Daily Living (ADLs) [24] and Lawton’s Instrumental ADLs [25] was self-reported or collected from proxies in case of cognitive impairment. Observed performance in Katz’ Basic ADLs as well as on the Barthel Index score [26] was measured at admission and at discharge. Data on the use of formal home care prior to admission was collected from patients and proxies in case of cognitive impairment.

### Assessment of cognitive and affective status

Cognitive status was assessed using Folstein’s Mini-Mental Scale Examination [27] (MMSE), with cognitive impairment defined as a score < 24/30. Affective status was examined based on the 15-item Geriatric Depression Scale [28] (GDS), which was performed only if patients scored 18 or more at MMSE. Affective impairment was defined as a GDS score of 6 or more. Complete data on mental (i.e., cognitive and affective) status was available for 612 subjects. Based on this information, patients were classified into one of the four following categories: 1) no cognitive or affective impairment; 2) affective impairment only; 3) cognitive impairment only; 4) combined cognitive and affective impairments.

### Measures of functional outcome, cost of rehabilitation, and discharge destination

For the purpose of this study, functional improvement was defined as any gain (i.e., at least a 5-point difference) on the Barthel Index score between admission and discharge from rehabilitation.

Information about length of rehabilitation stay and discharge destination (home, permanent nursing home admission, short stay in nursing home, readmission to acute care) was collected from the hospital administrative database.

In Switzerland, rehabilitation is billed on the basis of a daily fixed amount. Therefore, cost of rehabilitation stay was computed using length of rehabilitation stay and cost per day (US$ 736), as billed to the insurance carrier.

Patients discharged to their home were classified according to their functional status at discharge as functionally independent (i.e., Basic ADL score of 5 or 6) vs dependent (Basic ADL score < 5). The analysis of characteristics associated to discharge destination used a dichotomized outcome (i.e., home vs other discharge destination).

### Statistical analysis

Change between admission and discharge scores on the Barthel Index was computed using the effect size: i.e. the difference between the mean admission and discharge scores divided by the standard deviation of the mean admission score. This statistic provides information on the magnitude of change in the measure while accounting for its variability at admission, allowing an estimation of the clinical relevance of the change over time. An effect size of 0.80 or above is considered as large, while a value of about 0.5 is moderate, and a value under 0.2 is small [29].

To examine the associations between the explanatory variable defining cognitive and affective impairments and 1) functional improvement (yes vs no), as well as 2) discharge destination (home vs other destination), we first performed unadjusted logistic regression analyses. Then, two separate multivariable logistic regression models investigated the independent association between the explanatory variable and each specific outcome (i.e., functional improvement and discharge destination, respectively). Both models included the same adjustment variables, selected among patients’ characteristics available upon admission and significantly associated with the outcome, namely: age, gender, living arrangement, use of formal home care prior to admission, functional status prior to admission, comorbidity, and Barthel Index score at admission.

Data analyses were performed with Stata Data Analysis and Statistical Software (version 12).

## Results

### Characteristics of patients admitted to rehabilitation after a hip fracture

Table 1 shows the characteristics of the study population. The typical patient was an eighty-year old woman who was living alone prior to the fracture, and independent in most basic ADLs (85% with a score of 5 or 6). At admission to rehabilitation, mean Basic ADL was 2 ± 1 and average Barthel Index score was 53 ± 17. At admission, 8.2% had isolated affective impairment, 27.5% had isolated cognitive impairment, and 7.5% had combined impairments.

**Table 1:** Characteristics of patients admitted to rehabilitation after surgery for a hip fracture

Characteristics	Patients with hip fracture (*n* = 612)
Age (years, mean ± SD)	84.1 ± 6.9
Women (%)	78.3
Living alone prior to hospitalization (%)	67.2
Formal home care prior to hospitalization (%)	50.6
Number of comorbidities (mean ± SD)	6.9 ± 3.7
Instrumental^a^ ADL score prior to hospitalization (mean ± SD)	5.3 ± 2.5
Basic ADL^b^ score prior to hospitalization (mean ± SD)	5.3 ± 1.0
Basic ADL^b^ score at admission (mean ± SD)	2.1 ± 1.3
Barthel Index score ^c^ at admission (mean ± SD)	53.0 ± 17.2
MMSE^d^ score (mean ± SD)	24.1 ± 5.5
GDS score^e^ (mean ± SD)	3.0 ± 2.7
Mental status^f^:	
-No cognitive, no affective impairment (%)	58.3
-Affective impairment only (%)	8.2
-Cognitive impairment only (%)	27.5
-Combined cognitive and depressive impairments (%)	7.5

^a^ Lawton’s scale for instrumental activities of daily living [25]. Include ability to use telephone, shopping, food preparation, housekeeping, laundry, use of public transportation, and ability to handle medications and money. Scores range from 0 to 8, with higher scores indicating higher function

^b^ Katz’ Basic Activities of Daily Living [24]. Include bathing, dressing, using the toilet, transferring between bed and chair, maintaining continence, feeding. Scores range from 0 to 6, with higher scores indicating higher function

^c^ Barthel Index: score ranges from 0 to 100, with higher score indicating better mobility and functional performances [26]

^d^ Based on Folstein’s Mini Mental Status Examination [27]. Scores range from 0 to 30, with a score < 24/30 indicating cognitive impairment

^e^ Based on the Geriatric Depression Scale, short form (15 items) [28]. Affective impairment defined as a score of 6 or more

^f^ Based on score at the MMSE and GDS instruments, patients were classified as 1) no cognitive, no affective impairment; 2) affective impairment only; 3) cognitive impairment only; 4) combined cognitive and affective impairments

### Functional outcomes

Overall, 88.3% of the patients improved on the Barthel Index score during their rehabilitation stay, whereas 7.7% remained stable, and only 4.3% further declined. However, the proportion of patients with in-hospital functional improvement steadily declined according to the presence of mental impairment, from 91.2% in patients without any impairment, to 88.4% in patients with isolated affective impairment, 86.1% in patients with isolated cognitive impairment, and 73.8% in patients with combined impairments.

**Fig. 1:**
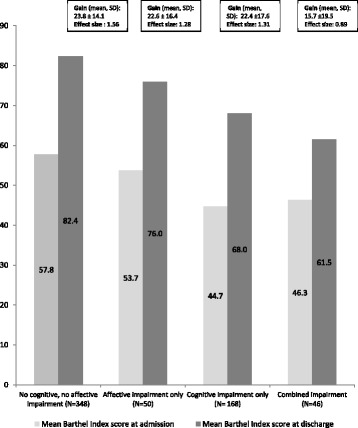
Evolution of Barthel Index score during rehabilitation stay in patients with hip fracture according to mental status at admission

As shown in Fig. 1, mean scores on Barthel Index at admission and at discharge were highest in patients without any mental impairment, whereas mean scores at discharge were lowest in patients with combined impairments. In-hospital functional improvement varied accordingly, with an average gain on the Barthel Index reaching 24 points in patients without any impairment, about 22 points in patients with either cognitive or affective impairment alone, and only 16 points in those with combined impairments. Nevertheless, each group achieved clinically and statistically significant gains, as indicated by effect size values all above 0.8.

Table 2 shows the results from bivariate and multivariate analyses investigating the relationship between mental impairment and functional improvement on the Barthel Index during rehabilitation. Compared to patients without impairment, those with combined impairments remained at lower odds of functional improvement after adjustment (adjOR: 0.40; 95%CI: 0.16–1.0; *p* = .049). Performance in Basic ADLs prior to admission (adjOR: 1.92, 95%CI: 1.48–2.48, *p* < .001) also remained significantly associated with increased odds of functional improvement, whereas higher scores at Barthel Index at admission were associated with decreased odds of further improvement (adjOR: 0.98, 95%CI: 0.96–0.99, *p* = .022).

**Table 2:** Results of bivariate and multivariate logistic regression analyses of the relationship between affective and cognitive impairment with functional improvement at rehabilitation discharge in patients with hip fracture

Characteristics	Improvement		Bivariate analysis			Multivariate analysis		
	Yes (*N* = 488)	No (*N* = 65)	UnadjOR	95%CI	*P*-value	AdjOR^a^	95%CI	*P*-value
Mental status:								
-No cognitive, no affective impairment (%)	91.2	8.8	reference	–	–			
-Affective impairment only (%)	88.4	11.6	0.74	0.27–2.02	.552	0.79	0.27–2.31	.661
-Cognitive impairment only (%)	86.1	13.9	0.60	0.33–1.09	.096	0.70	0.34–1.43	.336
-Combined cognitive and depressive impairment (%)	73.8	26.2	0.27	0.12–0.60	.001	0.40	0.16–1.00	.049
Age (years, mean ± SD)	83.9 ± 7.0	85.4 ± 6.9	0.98	0.94–1.01	0.164	0.97	0.93–1.01	.260
Women (%)	79.9	71.2	1.61	0.93–2.79	0.09	1.44	0.73–2.89	.292
Living alone prior to hospitalization (%)	68.0	53.5	1.84	1.12–3.05	0.02	1.54	0.81–2.93	.185
Formal home care prior to hospitalization (%)	47.9	58.8	0.64	0.38–1.07	0.09	0.75	0.41–1.38	.355
Number of comorbidities (mean ± SD)	6.8 ± 3.6	7.2 ± 3.7	0.97	0.91–1.03	0.33	1.01	0.93–1.10	.696
Basic ADL^b^ score prior to hospitalization (mean ± SD)	5.4 ± 0.4	4.5 ± 1.6	1.72	1.42–2.09	<.001	1.92	1.48–2.48	<.001
Barthel Index score^c^ at admission (mean ± SD)	53.2 ± 16.5	50.4 ± 24.5	1.01	0.99–1.02	*0.214*	0.98	0.96–1.00	.022

^a^ Logistic model including patient’s characteristics presented in the table

^b^ Katz’ Basic Activities of Daily Living [24]. Include bathing, dressing, using the toilet, transferring between bed and chair, maintaining continence, feeding. Scores range from 0 to 6, with higher scores indicating higher function

^c^ Barthel Index score used as a continuous variable (range: 0 to 100, with higher score indicating better mobility and functional performances) [26]

Cognitive impairment defined as a score < 24/30 at Folstein’s Mini Mental Status Examination (Scores range from 0 to 30, with higher score indicating higher cognitive function [27] Affective impairment defined as a score of 6 or more at Geriatric Depression Scale, short form [28]

### Discharge destination

Overall, the majority of patients returned back home after their rehabilitation stay. This proportion however varied according to the presence of mental impairment, with a steady decline from the group without any impairment (82.8%) to the one with both affective and cognitive impairments (45.7%).

**Fig. 2:**
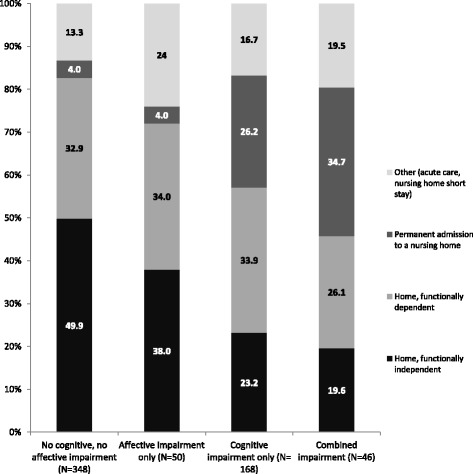
Discharge destination in patients with hip fracture according to mental status at admission

The proportion of patients discharged home who were functionally independent also varied according to mental status (Fig. 2), decreasing from almost 50% in patients without any impairment to less than 20% among those with combined impairments. Similarly, nursing home admission occurred quite rarely in patients without any impairment or with isolated affective impairment (4.0% in both cases), whereas 26.2% of patients with cognitive impairment only, and 34.7% of those with combined impairments were discharged to a nursing home for long term care.

Table 3 shows the results from bivariate and multivariate analyses investigating the relationship between affective and cognitive impairments with discharge destination*.* In bivariate analyses, compared to patients with other destination, those discharged home were less likely to have cognitive impairment, with or without affective impairment. After adjustment, isolated cognitive impairment (adjOR: 0.57; 95%CI: 0.35–0.92; *p* = .023), and combined affective and cognitive impairments (adjOR: 0.31; 95%CI: 0.15–0.66; *p* = .002) both remained associated with decreased odds of returning home. Older age (adjOR: 0.95 per additional year, 95%CI: 0.92–0.98, *p* = .003) also reduced the odds of home discharge, whereas better function on the Barthel Index score at admission was associated with higher odds of being discharged home (adjOR: 1.05, 95%CI: 1.03–1.06, *p* < .001).

**Table 3:** Results of bivariate and multivariate analyses of the relationship between affective and cognitive impairment with discharge destination after rehabilitation in patients with hip fracture

Characteristics	Destination		Bivariate analysis			Multivariate analysis		
	Home (*N* = 441)	Other (*N* = 171)	UnadjOR	95%CI	*P*-value	AdjOR^a^	95%CI	*P*-value
Mental status:								
-No cognitive, no affective impairment (%)	82.8	17.2	1.0	–	–			
-Affective impairment only (%)	72.0	28.0	0.54	0.27–1.05	.071	0.54	0.26–1.14	.107
-Cognitive impairment only (%)	57.1	42.9	0.28	0.18–0.42	<.001	0.57	0.35–0.92	.023
-Combined cognitive and depressive impairment (%)	46.6	54.4	0.18	0.09–0.33	<.001	0.31	0.15–0.66	.002
Age (years, mean ± SD)	83.3 ± 7.0	86.2 ± 6.5	0.94	0.91–0.96	<.001	0.95	0.92–0.98	.003
Women (%)	79.6	72.2	1.51	1.02–2.21	.037	1.40	0.83–2.37	.205
Living alone prior to hospitalization (%)	67.2	62.3	1.24	0.87–1.76	.233	.84	.51–1.38	.492
Formal home care prior to hospitalization (%)	45.3	63.8	0.47	0.33–0.67	<.001	0.79	0.50–1.25	.313
Number of comorbidities (mean ± SD)	6.7 ± 3.6	7.6 ± 3.8	0.93	0.89–0.98	.002	0.97	0.92–1.03	.304
Basic ADL^b^ score prior to hospitalization (mean ± SD)	5.4 ± 0.9	4.8 ± 1.3	1.60	1.36–1.88	<.001	1.14	0.93–1.41	.198
Barthel Index score^c^ at admission (mean ± SD)	56.6 ± 15.7	40.8 ± 18.2	1.06	1.04–1.07	<.001	1.05	1.03–1.06	<.001
Barthel Index score^c^ at discharge (mean ± SD)	81.6 ± 14.7	52.6 ± 22.9	1.07	1.06–1.09	<.001	–	–	–

^a^ Logistic model including patient’s characteristics available upon admission, presented in the model

^b^ Katz’ Basic Activities of Daily Living [24]. Include bathing, dressing, using the toilet, transferring between bed and chair, maintaining continence, feeding. Scores range from 0 to 6, with higher scores indicating higher function

^c^ Barthel Index score used as a continuous variable (range: 0 to 100, with higher score indicating better mobility and functional performances) [26]

Cognitive impairment defined as a score < 24/30 at Folstein’s Mini Mental Status Examination (Scores range from 0 to 30, with higher score indicating higher cognitive function) [27] Affective impairment defined as a score of 6 or more at Geriatric Depression Scale, short form [28]

### Length of stay and cost

**Fig. 3:**
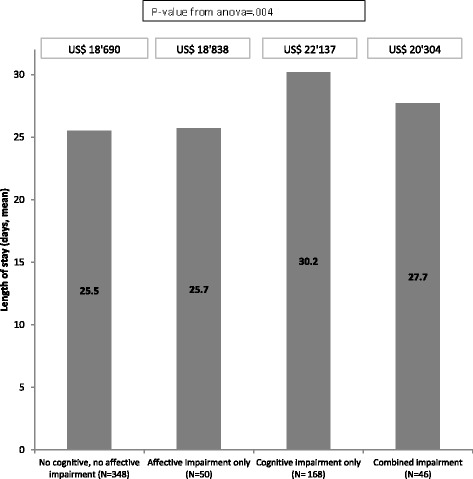
Mean length of rehabilitation stay and costs according to mental status at admission

As shown in Fig. 3, average length of rehabilitation stay and costs differed across the four groups of patients (*P*-value from analysis of variance = .004). Length of stay was shortest in patients without any impairment (25.5 days, corresponding to about US$19′000, as billed for reimbursement by the health care insurance) whereas patients with cognitive impairment had longest stays and highest corresponding average cost (US$ 22′617).

## Discussion

This work gives more insight into the interplay between affective and cognitive impairments on functional improvement, discharge destination, and costs in older hip fracture patients admitted to rehabilitation. It extends previous findings by outlining that isolated affective or cognitive impairment both show a marginal association with poorer rehabilitation outcomes [20, 26, 30–32]. Although this negative impact might appear relatively limited, it is still likely to prevent a significant proportion of patients to achieve their full rehabilitation potential. Indeed, about one in three patients had either cognitive or affective impairment, a proportion consistent with studies in similar settings [11, 15, 22].

An important and original contribution of this study is to highlight the complexity of the relationship between mental impairments and the selected functional and discharge outcomes. Indeed, results of analyses about functional improvement almost suggest a dose-response relationship across groups of patients, those with combined impairments showing notably worse outcome. However, the current work simultaneously indicates that functional gain was clinically significant even in patients impaired in both affective and cognitive status, a finding that extends results from previous studies in similar populations [12, 19, 33–37]. A practical and important implication of these results is that it would be unjustified to deny rehabilitation in these patients.

As affective and cognitive problems are still often overlooked [30] despite their high prevalence among hip fracture patients, results also strongly support the rationale for systematic cognitive and affective screening, followed by further management. In addition, results also indicate the need to further developing innovative rehabilitation strategies to improve these patients’ engagement and motivation into the rehabilitation process as well as to better meet their specific needs [31, 32, 38]. Indeed, significant functional improvement might still be achieved in patients with moderate to severe cognitive problems, because performing motor training uses procedural memory, which remains preserved even in later stages of dementia [39]. Their functional gain might be further optimized by modifying specific modalities of rehabilitation, such as frequency, intensity, duration or particular components of sessions [31]. Similarly, hip fracture patients with affective impairment should be offered adapted stationary rehabilitation programs that include specific psychological support strategies such as motivational interventions to enhance their functional recovery [22, 40].

A specific contribution of this study is also to emphasize the differential effect of cognitive and affective impairments on the selected outcomes: while cognitive impairment was clearly associated to a higher rate of nursing home admission, with an additional increase when both affective and cognitive impairments were present, affective impairment alone did not significantly affect the likelihood of being institutionalized. This finding contrasts with some other studies identifying depressive symptoms as a risk factor for nursing home admission [18, 41]. However, these previous studies were performed in heterogeneous settings and did not always control for potential confounders [16].

### Strengths and limitations

A clear strength of this study is the relatively large sample of patients included. An additional advantage is the use of robust and reliable measures of function that detect clinically significant changes over time, thus avoiding overestimating rehabilitation benefits. Similarly, effect size calculation further strengthens the clinical relevance of observed benefits. Finally, costs data that are an original contribution of this study represents real costs as billed to the insurance carriers even though they were not based on analytic costs.

A limitation of this study was that cognitive and affective status stemmed from screening instruments, not from diagnostic workup. Also, no information was collected on further interventions following positive screening for affective impairment. However, the relatively short follow-up period, restricted to the length of the rehabilitation stay, limits the potential for improvement in affective status as a consequence of therapeutic interventions. This short follow-up period is also another potential limitation, but it corresponds to the length of rehabilitation, as most patients do not further receive physical therapy when discharged to their home. Finally, another restriction was that participants were community-dwelling older patients admitted to a single rehabilitation center following surgery for hip fracture, thus precluding the generalization of our findings to other settings and populations, such as nursing home residents with hip fractures. Nevertheless, this sample appears quite similar to those enrolled in similar studies of community-dwelling patients [2, 7, 33, 42].

## Conclusions

This study in older patients admitted to rehabilitation after a hip fracture highlights that affective and cognitive impairments have both independent, and synergistic negative effects on functional outcome and discharge destination. However, patients with combined affective and cognitive impairments still achieved significant functional improvement, even though its magnitude was reduced. As a perspective, the development of specific, better adapted rehabilitation strategies targeting these patients might help to optimize their functional recovery.

## Abbreviations

adjOR: Adjusted odds ratio
ADLs: Activities of daily living
CI: Confidence interval
GDS: Geriatric depression scale
MMSE: Mini-mental state examination
OR: Odds ratio
US$: United States dollar
